# Using the FIB-4, automatically calculated, followed by the ELF test in second line to screen primary care patients for liver disease

**DOI:** 10.1038/s41598-024-62549-3

**Published:** 2024-05-28

**Authors:** Denis Ouzan, G. Penaranda, M. Jlaiel, H. Joly, J. Corneille

**Affiliations:** 1 Département d’Hépato-Gastroentérologie, Institut Arnault Tzanck, 06700 Saint-Laurent-du-Var, France; 2 Réseau ville hôpital hépatite C Cote d’Azur (RHeCCA), Nice, France; 3 Laboratoire Alphabio-Biogroup, Marseille, France; 4 Hôpital Européen, Marseille, France; 5 Laboratoire Bioesterel-Biogroup, Mandelieu-la-Napoule, France; 6 Laboratoire Bioesterel-Biogroup, Mougins, France

**Keywords:** Liver fibrosis, Non-invasive tests, Fibrosis-4 test, ELF, Screening, General practice, Biomarkers, Health care

## Abstract

The objective of our work was to evaluate the screening of hepatic fibrosis in primary care using the FIB-4 score, automatically calculated. When the FIB-4 was ≥ 1.3, it was defined as positive, and ELF Test was performed. FIB-4 positivity was confirmed when ELF Test was ≥ 9.8 indicating an advanced fibrosis. Among the 3427 patients included, 869 (25%) had a positive FIB-4 score, 784 (22.5%) at intermediate (FIB-4: 1.3–2.67), and 85 (2.5%) at high risk of fibrosis (FIB-4 > 2.67). 509 (59%) of the FIB-4 positive were confirmed by the ELF Test. The percentage of confirmation was significantly higher in patients over 65 years (83 vs. 57%), with FIB-4 > 2.67 (80 vs. 56%), BMI > 25 (47 vs. 37%), and diabetes (24 vs. 14%), p = 0.001). In patients without known liver disease (92%), the practitioner identified a cause of disease in 27% of cases: mainly NAFLD and alcohol. Liver fibrosis was suspected on FIB-4 in 25% of patients in primary care. The ELF Test, performed as a second-line, improves the screening of liver fibrosis, particularly for FIB-4 intermediate results. A positive FIB-4 test allows physicians to recognize a liver disease, providing an opportunity for timely intervention.

**Clinical trial registration**: Comité de protection des personnes du sud-ouest et outre-mer SI18.00832.201865-MS04-IDRCB 2018-A01571-54.

## Introduction

Chronic liver disease (CLD) can lead to a progressive accumulation of fibrosis in the liver, potentially progressing to cirrhosis and hepatocellular carcinoma. The burden of CLD worldwide is substantial, accounting for approximately 2 million deaths annually due to cirrhosis and HCC. The primary causes are non-alcoholic fatty liver disease (NAFLD), alcohol-related liver disease (ALD), and viral hepatitis, among other less common causes^1–6^. The prognosis and management of CLD patients are closely tied to liver fibrosis, making treatment of the underlying CLD critical to prevent further fibrosis progression to cirrhosis and its complications^3–6^.

While liver biopsy was once the reference procedure for evaluating liver fibrosis, its invasive nature makes it unsuitable as a first-line procedure. Blood tests and liver stiffness measurements by transient elastography have been developed as non-invasive alternatives, gradually replacing biopsy^7^. However, the high cost of accurate blood fibrosis tests (such as Fibrosure, Fibrometer, ELF (Enhanced Liver Fibrosis) Test) restricts their widespread use, and liver elastometry is only available in specialized centers^8–11^.

Other scores, which are simple and usable in current medical practice, rely on commonly measured biomarkers: FIB-4, APRI, NAFLD Fibrosis Score^12–14^. FIB-4 outperforms other calculations in identifying patients with a low probability of advanced fibrosis^15–17^. High or indeterminate FIB-4 scores are associated with an increased incidence of severe liver disease in primary care patients without known CLD^18^, and high FIB-4 values have also been associated with liver-related outcomes in population-based studies^18,19^. Therefore, FIB-4 is recommended as a first-line fibrosis assessment for general practitioners and endocrinologists in patients with metabolic or alcoholic co-factors, helping identify those who need referral to a specialist liver clinic^17,20^.

In a community study, 2-tier testing with FIB-4 followed by ELF Test in patients with indeterminate FIB-4 results improved the detection of advanced fibrosis fourfold and reduced unnecessary referrals by 88%^21^. A more recent study show that the sequential combination of FIB-4 followed by ELF in intermediate cases minimizes the number of futile referrals, and saves cost, making this a promising referral strategy^22^. Therefore, ELF Test seem to be a good option for secondary risk assessments after a FIB-4 score, especially when elastography is not available^20–22^.

In practice, most patients with CLD are seen and managed by general practitioners. These practitioners often encounter challenges in evaluating a liver disease that remains asymptomatic for many years with normal routine diagnostic tests. Additionally, general practitioners have limited access to the best noninvasive liver fibrosis tests. As a result, many patients with progressive fibrosis go undiagnosed, with CLD only identified when they have advanced to the stage of cirrhosis^18^. We demonstrated in a previous study that the FIB-4 index can detect liver fibrosis in primary care and identify potential causes of liver disease in patients without known CLD^23^. Our work convinced all the clinical laboratories in the French Alpes Maritimes area (120 laboratories) to systematically calculate FIB-4 whenever transaminases and platelets were prescribed, starting from October 1, 2020. Early detection and management of CLD could then be facilitated by using a systematic FIB-4 score calculation in primary care.

## Objectives and methods

The main objective of our study was to assess the relevance of fibrosis screening via FIB-4 score, automatically calculated by the clinical laboratory in adults consulting in general medicine, outside of emergency or acute pathologies. Secondary objectives were to evaluate the impact of this automatic FIB-4 calculation on patient management and care pathways by general practitioners, and to propose a management algorithm for individuals recognized as FIB-4 positive in primary care through systematic FIB-4 calculation.

Over a six-month period from March to September 2022, FIB-4 scores were prospectively calculated for all consecutive patients seen by 15 general practitioners outside of emergency. These patients gave their informed consent to the general practitioner prior to FIB-4 completion. The inclusion criteria were adult primary care patients for whom the general practitioners had prescribed platelet and transaminase assays, prompting an automatic FIB-4 calculation. The FIB-4 thresholds used were: low risk if < 1.30, intermediate risk between 1.3 and 2.67, and high risk if > 2.67. When the FIB-4 score was ≥ 1.3, it was deemed positive, and a confirmatory ELF Test was systematically performed on the same sample used for FIB-4 calculation.

The ELF Test, a proprietary blood test, measures three elements involved in matrix turnover: hyaluronic acid (HA), tissue inhibitor of metalloproteinase-1 (TIMP-1), and N-terminal procollagen III peptide (PIIINP)^10^. A positive FIB-4 score was confirmed when the second-line ELF Test was ≥ 9.8. An ELF score of ≥ 9.8 reliably identifies patients at increased risk of advanced fibrosis and progression to cirrhosis and liver-related clinical events, while an ELF test ≥ 11.3 indicates severe fibrosis or cirrhosis^10^.

The results of the FIB-4 and ELF tests were relayed to the general practitioners, who anonymously provided clinical medical information to the investigation team for all FIB-4 positive individuals. The management of FIB-4 positive patients was left to the discretion of the general practitioner. This information included known or unknown liver disease and the main risk factors for liver disease (overweight, diabetes, alcohol consumption exceeding 100 g/week, hepatitis B or C infection). If liver disease was previously unknown, the general practitioner specified whether a positive FIB-4 result led them to suspect CLD, defined its cause (NAFLD, alcohol, virus, other), and determined if specialized advice was needed. The management of FIB-4 positive patients and the request for specialized advise was left to the discretion of the general practitioner. In this study, the ELF score was considered the reference test for detecting hepatic fibrosis.

### Statistical methods

Quantitative data were reported as mean, standard deviation (SD), and range (ie. difference between highest and lowest value); qualitative data were reported as frequency and percentage. Qualitative data were compared among groups using the Chi-squared test or the Cochran-Armitage test for trend, respectively, for the comparison between two binary parameters, and to assess whether a trend exists between a binary dependent variable and an independent ordinal scale variable with more than two ordered categories. All calculations were performed using SAS V9.4 software (SAS Institute Inc., Cary, NC).

### Ethics

All procedures were performed in accordance with relevant guidelines. The study protocol was approved by the Ethics Committee of Sud-Ouest et Outre-Mer IV, France (N° SI 18.00832.201865-MS04). The study was registered on N° EudraCT /ID-RCB 2018-A01571-54.

## Results

A total of 3427 patients were included; the mean age was 56 years (SD 17) [Range 80], and 35% were older than 65 years. An increased aspartate aminotransferase (AST) rate was found in 10% of patients, alanine aminotransferase (ALT) in 7%, and a decrease in platelet count < 150,000/mm^3^ in 2% (Table 1). Among the 3427 consecutive patients included, 869 (25%) had a positive FIB-4 score ≥ 1.3. Of these, 784 (22.5%) were at intermediate risk (FIB-4 between 1.3 and 2.67) and 85 (2.5%) were at high risk of fibrosis (FIB-4 > 2.67). FIB-4 positivity was significantly associated with age over 65 years, AST levels > ULN, AST/ALT ratio > 1, AST/ALT ratio > 1 in patients with ALT levels > ULN, and platelet levels < 150,000/mm3 (p < 0.001) (Table 2). The ELF Test was performed in the 869 patients with FIB4 ≥ 1.3. Among these, confirmation by the ELF Test (≥ 9.8) was observed for 509 (59%) patients (Table 3). Confirmation by the ELF test was observed in 80% of the patients with high-risk FIB-4, and 56% of those with intermediate-risk FIB-4 (p < 0.0001) (Table 3).

**Table 1:** Characteristics of the 3427 patients.

Caracteristics	N = 3427
Age—mean (Sd) [Range] (years)	56 (17) [80]
Age—N (%)	
< 65	2217 (65%)
≥ 65	1210 (35%)
≥ 70	877 (26%)
≥ 80	216 (6%)
ALT > ULN—N (%)	253 (7%)
AST > ULN—N (%)	353 (10%)
Platelets < 150,000—N (%)	59 (2%)

ALT, alanine aminotransferase; AST, aspartate aminotransferase; ULN upper limit of the normal.

**Table 2:** Characteristics of patients according to the FIB4 thresholds.

FIB-4	FIB4 < 1.3 (n = 2558)	FIB4 [1.3, 2.67] (n = 784)	FIB4 > 2.67 (n = 85)	P-Value*
Age—mean (Sd) [Range] (years)	51 (17) [76]	70 (10) [62]	76 (11) [48]	< 0.0001**
Age—N (%) (years)				
< 65	1976 (77%)	227 (29%)	14 (16%)	< 0.0001
≥ 65	582 (23%)	557 (71%)	71 (84%)	
ALT > ULN—N (%)	187 (7%)	46 (6%)	20 (24%)	0.0421
AST > ULN—N (%)	167 (7%)	141 (18%)	45 (53%)	< 0.0001
Platelets < 150,000—N (%)	6 (< 1%)	24 (3%)	29 (34%)	< 0.0001
AST/ALT ratio > 1—N (%)	1122 (44%)	541 (69%)	69 (81%)	< 0.0001
AST/ALT ratio > 1 in patients with ALT > ULN—N (%)	1/187 (< 1%)	4/46 (9%)	11/20 (55%)	< 0.0001**

ALT, alanine aminotransferase; AST, aspartate aminotransferase; ULN upper limit of the normal.

*Cochran-Armitage test; **Kruskal–Wallis test.

**Table 3:** Results of ELF score according to FIB-4 risk category.

FIB-4 > 1.3 N = 869/3427 (25%)	N (%)	FIB-4 [1.3–2.67] 784/3427 (22.5%)	FIB4 > 2.67 85/3427 (2.5%)	P-Value*
ELF—N (%)				< 0.0001
≥ 9.8	509 (51%)	441 (56%)	68 (80%)	
≥ 11.3	65 (8%)	47 (6%)	18 (21%)	

*Cochran-Armitage test.

Clinical information was obtained for 755 out of 869 (87%) FIB-4 positive patients. The percentage of confirmation by the ELF Test was significantly higher in patients over 65 years (84 vs 58%, p < 0.0001), in patients with FIB-4 ≥ 2.67 (80 vs 56%, p < 0.0001), those with BMI > 25 (47 vs 37%, p = 0.012), and in those with diabetes (24 vs 14%, p = 0.001), but not in those with excessive alcohol consumption (14 vs 15%, p = 0.9283) (Table 4). Only 8% of the patients were known to have liver disease. Confirmation by the ELF Test was significantly more frequent in subjects with known liver disease than in those without. Among FIB-4 positive patients without known liver disease, who represented the majority of patients, general practitioners defined a cause for 27% of cases (NAFLD, 67%; alcohol, 23%; FLD+ alcohol, 9%; others, 5%) (Table 5), with no difference observed according to confirmation by the ELF Test. They sought specialized advice for half of the patients for whom they could suggest a cause of liver disease (Table 5).

**Table 4:** Univariate analyses of factors associated with FIB4 confirmation by ELF test.

	FIB4 ≥ 1.3 followed in second line by an ELF TEST		P-Value*
	ELF < 9.8 (N = 301)	≥ 9.8 (N = 454)	
Age—N(%)			< 0.0001
< *65*	127 (42%)	73 (16%)	
≥ *65*	174 (58%)	381 (84%)	
Gender—N (%) (N = 753)			0.4606
Male	138 (46%)	222 (49%)	
Female	161 (54%)	232 (51%)	
BMI > 25—N (%)	113/299 (38%)	211/446 (47%)	0.0102
Diabetes—N (%)	41 (14%)	108 (24%)	0.0006
Alcohol—N (%)	45 (15%)	65 (14%)	0.8284

BMI, body mass index; ELF, enhance liver fibrosis.

*Chi-squared test.

**Table 5:** Information provided by general practitioners in patients with FIB-4 > 1.3 and unknown liver disease.

Patients with FIB-4 >1.3 and unknown liver disease		N: 688	Specialized advice
Cause evoked—N (%)		188 (27%)	90
NAFLD		114 (61%)	
NAFLD + Alcohol		19 (10%)	
Alcohol		46 (25%)	
Other		9 (5%)	

NAFLD, nonalcoholic fatty liver disease.

## Discussion

A decade has passed since non-invasive markers of liver fibrosis emerged, ranging from simple scores like APRI, FIB-4, NAFLD Fibrosis Score to more intricate tools such as the ELF Test, Fibrometer, Fibrosure, Transient Elastography. Broadly speaking, their use remains largely confined to secondary specialist liver care. Even with the availability of tools to assess liver fibrosis severity and treatments for liver disease, early diagnosis before the onset of liver complications is still too rare^18^. FIB-4 is the most validated among the simple tests for fibrosis^16,17,20^. It has been recommended for use in primary care and diabetology settings for subjects with risk factors for liver disease, such as metabolic issues or alcohol consumption^17,20^.

Yet, a recent study revealed that general practitioners are not familiar with non-invasive markers of liver fibrosis, particularly FIB-4^24^. This study suggests that a lack of awareness of CLD among general practitioners and diabetologists might contribute to this shortfall in the diagnosis of advanced CLD.

According to a study reported in primary care on FIB-4, 20% of subjects with a high risk of fibrosis, as defined by an initial FIB-4 > 2.67, encountered a serious liver event (cirrhosis, cancer, transplantation) after an average follow-up of 8 years. Half of them were discovered to have liver disease at the time of the event^18^. Therefore, FIB-4 could potentially allow for the detection of liver disease before the onset of serious events. Most recent recommendations propose that the FIB-4 score should be used as a first-line test for screening liver fibrosis in patients with metabolic or alcoholic co-factors, followed by a more accurate test such as the Fibroscan or the ELF Test^17,20^. The sequential combination of FIB-4 followed by ELF in intermediate cases minimizes the number of futile referrals in patients with NAFLD, and saves cost, making this a promising referral strategy, which improves resource use and benefits patients^21^. Recent European guidelines advocate for a three-tiered approach, beginning with FIB-4, followed by Fibroscan, and finally, a specialized blood test^17^.

In a prospective study we conducted on primary care patients without known liver disease, we used FIB-4 to screen for significant liver fibrosis. This revealed that a positive FIB-4 score led general practitioners to suspect an unrecognized liver disease in two-thirds of the cases^23^. Following this study, automatic calculation of FIB-4 was implemented in the 120 clinical laboratories of the Alpes Maritimes area in France. The goal of our study was to evaluate this automatic calculation in general practice. We achieved this by performing a confirmatory ELF Test for all subjects with a positive FIB-4, using the same sample that was used to calculate the FIB-4. The aim was to define a decision-making algorithm for the discovery of a positive FIB-4 in primary care.

In a six months period, 25% of the subjects seen by a group of general practitioners had FIB-4 scores over 1.3. Above 1.3, the distribution of the zones at risk of hepatic fibrosis was consistent with usual findings: 2.5% for the high-risk zone and 22.5% for the intermediate-risk zone^18,21,23,25^. Elevated transaminases, which indicate possible liver disease, were found in 7–10% of cases, or about one-third of what was observed with FIB-4.

**Figure 1:**
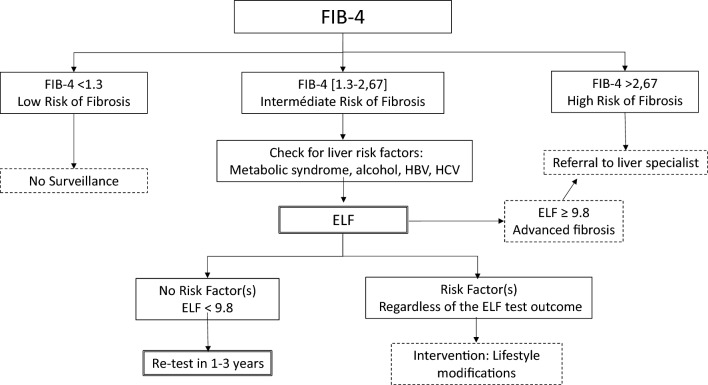
Management algorithm for subjects recognized as FIB-4 positive in primary care by the systematic calculation of FIB-4.

A positive FIB-4 score was significantly associated with specific liver abnormalities, such as elevated AST, decreased platelet count, and an AST/ALT ratio > 1 in patients with elevated ALT. This confirms FIB-4 as an indicator of liver disease. Subjects in the high-risk zone (FIB-4 > 2.67) and those over 65 years old were confirmed by the ELF Test in 80% of cases. This calls into question the necessity of confirming FIB-4 in these patient subgroups. Subjects in the high fibrosis risk area more often had an ELF score ≥ 11.3 signaling cirrhosis and those with ALT levels > ULN more frequently showed an AST/ALT ratio > 1, indicative of severe fibrosis or cirrhosis^14,26^. These findings underscore the high risk of severe fibrosis lesions in subjects with FIB-4 > 2.67. Thus, we recommended that general practitioners seek specialist advice for all subjects in the high-risk fibrosis zone and in those with an ELF test ⪖ 9.8 (Fig. 1).

The FIB-4 intermediate risk zone is much broader but deserves consideration as it corresponds to a 56% ELF Test confirmation rate, which has been found in other studies^21,22^. Confirmation is more frequent when a hepatic risk factor such as overweight or diabetes is present. In general, subjects with a risk factor should be confirmed as a priority. Surprisingly, excessive alcohol consumption did not link to more frequent confirmation, probably due to underestimation, but requires intervention from general practitioners, as does overweight, as soon as the FIB4 score is positive, regardless of confirmation (Fig. 1).

92% of the patients screened with a positive FIB-4 test had unknown liver disease. However, the general practitioner was able to recognize a liver disease in 27% of the cases of FIB-4 positive, primarily NAFLD (63% of the cases) (Table 5). Despite the lower percentage compared to our previous study, it confirms that a positive FIB-4 found in primary care can serve as an early warning, allowing for the suspicion and potential diagnosis of liver disease, independently of the fibrosis, which could be absent or minimal and thereby acting as a true marker of liver disease^23^. To support this hypothesis, a cause of liver disease has been suggested in subjects with a positive FIB-4 with no difference between patients confirmed or not by the ELF Test.

The positivity of the FIB-4 may also increase the patient's awareness of a liver disease risk factor (overweight and excessive alcohol consumption), providing the general practitioner informs the patient about the significance of this FIB-4 score.

There is agreement on the need to educate general practitioners on the importance of fibrosis screening. The introduction of an automatic calculation of FIB-4, which can be easily implemented by clinical laboratories, could initiate and encourage general practitioners to detect and manage chronic liver diseases earlier.

This study has some limitations. The first limitation is that general practitioners provided medical information retrospectively after receiving the results of the FIB-4 and ELF tests. The second is that the ELF Test was performed only among the 869 patients with FIB4 ≥ 1.3; thus, 75% of the cohort did not have ELF assessment. However, it is well known that a FIB-4 score < 1.3 is associated with a strong negative predictive value for advanced liver fibrosis^17^. The third limitation is that, although clinical information was retrieved for a large majority of patients (n = 755), we do not have medical information for patients with FIB-4 < 1.3. Therefore, we are unable to compare the percentage of patients with hepatic risk factors according to the FIB-4 threshold of 1.3. Lastly, our study concedes a limitation in the identification of potential liver disease causes, as these are determined subjectively by the attending physician without any objective evidence to support the diagnosis.

## Conclusion

Liver fibrosis was suspected by FIB4 score automatically calculated in 25% of patients who consulted a general practitioner and was confirmed by ELF test in 59% of cases. The percentage of confirmation by the second line ELF test was significantly higher in patients with a high-risk FIB-4 score ≥ 2.67, in patients over 65 years (both groups for which a confirmation is questionable) and in those with a risk factor of liver disease overweight or diabetes, who require an intervention and need to be confirmed in priority. High-risk FIB4 ≥ 2.67 patients should be referred for specialist advice. In patients without known liver disease, the FIB-4 allow the general practitioner to recognize a liver disease in nearly one third of cases, mainly NAFLD. Thus, automatic calculation of the FIB-4 may represent an initial step to enhance the recognition and management of liver disease in the general population. The ELF test performed as a second-line test improves the screening of hepatic fibrosis by FIB-4, in particular for FIB-4 intermediate results, in patients under 65 years of age, who have a risk factor of liver disease.

## Data Availability

The datasets used and/or analyzed during the current study available from the corresponding author on reasonable request.

## Abbreviations

CLD: Chronic liver disease
NAFLD: Non-alcoholic fatty liver disease
FLD: Fatty liver disease
ALD: Alcohol–related liver disease
ULN: Upper limit of the normal
